# Foreign

**Published:** 1841-05-15

**Authors:** 


### FOREIGN.

### Sir Astley Cooper.

Sir Astley Cooper was born August 23d, 1768, at Brooke, in Norfolk, and died at his house in London, Feb. 12, 1841, being in his seventy-third year. He was the son of a clergyman in the church of England—Dr. Samuel Cooper, of Yarmouth, who married Miss Bransby, of Shottisham, a lady of talent, herself an authoress, and connected with the family of Paston,—a circumstance which led to this name also being given to Sir Astley.

It has often been remarked that some circumstance, apparently accidental, has tended to influence the future career of those concerned; and an anecdote is told of Sir Astley which, if true, seems to bear out this idea. It is said that when a boy he saw a lad fall from a cart, and tear his thigh in such a manner as to wound the femoral artery; our young hero immediately took his handkerchief, applied it round the thigh, and twisted it so tightly as to control the bleeding till further assistance could be procured.

At the age of fourteen he was removed to Yarmouth, where he was soon after apprenticed to Mr. Turner, a general practitioner in that town. In this situation, however, he remained but a very short time ere he proceeded to London, to be under the care of his uncle, Mr. W. Cooper, one of the surgeons of Guy's Hospital, by whom, however, he was in a few months transferred, at his own request, to Mr. Cline, then in the height of his reputation at St. Thomas's. On the the completion of the ordinary course of study, and while yet in his apprenticeship, he became demonstrator of anatomy to his distinguished master; and in 1791 he began to lecture on surgery, giving the first regular course on that subject ever delivered in London; as, anterior to this time, what surgery was given constituted but a collateral branch of the anatomical course.

In 1787 he spent the winter in attendance on the medical classes in Edinburgh; and in 1792 he went to Paris, having previously married Miss Cock, a connection of Mr. Cline, by whom he had one daughter—his only child, who died in her second year.

During his residence in the French capital he attended chiefly to the lectures and practice of Desault, at that time the most distinguished surgeon in France, and of whose instructions he entertained a very high opinion. Here Sir Astley was present during some of the horrors of the revolution, and among other things was a witness of the celebrated attack upon the Tuilleries, and the massacre of the Swiss Guards, on 10th of August, 1792.

On his return from Paris, Sir Astley permanently settled in practice. In 1800 he became surgeon to Guy's Hospital—an appointment which he retained till 1826, when he was created consulting surgeon.

Sir Astley's first house was in Jeffrey's Square, St. Mary Axe—not a very fashionable part of the town, certainly, but where he was content to sojourn not less than six years. He then removed to New Broad Street, where he remained for seventeen years in very extensive practice, and in daily increasing reputation. His income was certainly at one time greater than that of any other medical man of the present day, having, we believe, on more occasions than one, exceeded £20,000 within a year. He also received some very large fees, among which not the least remarkable was that of a thousand guineas thrown at him in his night-cap by a patient whom he had cut for the stone—an anecdote which we heard the deceased tell with no small animation, on retiring from a patient upon whom he had just performed the same operation, and who had likewise in his agony flung his cap at the surgeon, but without its containing on this occasion the cheque which gave so much force to the original incident.

In 1815, when at the height of his reputation, he removed to Spring Gardens; and he was one of the few with whom the migration from the city to the west end has proved fully successful. A few years afterwards he was employed professionally by George IV. to remove a small tumor from the scalp—an operation which he performed with all his wonted coolness and dexterity.*

Soon after this (in 1821) he was created a baronet, the patent extending to his nephew and namesake, Astley Paston Cooper, fourth son of his brother—the present holder of the title. From this time till 1827 he continued to enjoy an extensive practice, and to make a very large income. He then, in the full zenith of his fame, voluntarily retired into the country to enjoy the riches he had accumulated, and spend the remainder of his days in the dignified repose of a country gentleman. But Sir Astley was not made for the *otium cum dignitate*, and a very short time saw him back again in the metropolis, where, on more than one occasion, he publicly referred to the period of his seclusion, and declared that if he had remained idle he should certainly have hanged himself. His nephew, Mr. Bransby Cooper, having been installed in his old residence in New Street, Spring Gardens, Sir Astley took a house in Conduit Street, where he gave a series of *conversazioni*, which were attended by nearly all the medical world in London, and which were intended apparently to convince his brethren of the reality of his return. He brought with him his great name and umblemished reputation, but never had, and probably never desired to have, the same immense business as before his temporary retirement; others, of scarcely inferior note, had gained possession of, and retained a considerable portion of what had before been almost exclusively his own.

His first wife having died in 1827, he married Miss Jones, of Cardiganshire, the following year, and from this time used to reside some portion of each season on his estate in the country. He also visited Paris more than once, and received marked attention from M. Dupuytren.

We have already mentioned that Sir Astley began to lecture on surgery at an early age (in 1791,) and he continued to do so till 1826. We attended his course somewhat more than twenty years ago, and our recollections vividly remind us of one who carried conviction to those who heard him, that he was a master of his art. He was not particularly studious of elegance, but his language was always fluent and clear; his ideas were precise, and his meaning never doubtful; the more important points of his subject always had their due prominence, and were deeply impressed on his hearers.

As an operator we need scarcely say that he was bold, rapid, and skilful, almost without parallel,—qualities which tended greatly to enhance his own reputation, and to heighten the character of English surgery. We well remember having been present at a conversazione at M. Maunoir's in 1817, attended by most of the scientific men in Geneva, when the fact was communicated of Sir Astley having cut down upon and tied the aorta in the living subject; nor shall we readily forget the expressions of admiration, not quite unmingled with consternation, with which the announcement was received.

Probably no surgeon of ancient or modern times has enjoyed a greater share of reputation during his life than has fallen to the lot of Sir Astley. The old and new world has alike rung with his fame; and, perhaps, we cannot give a better example of this than one to which we remember having alluded on a former occasion —we mean, the fact of his signature being received as a passport among the mountains of Biscay, by the wild followers of Don Carlos. A young English Surgeon, seeking for employment, was carried as a prisoner before Zumalacarregui, who demanded what testimonials he had of his calling or his qualifications? Our countryman presented his diploma of the College of Surgeons; and the name of Astley Paston Cooper, which was attached to it, no sooner struck the eye of the Carlist leader, than he at once received his prisoner with friendship, and appointed him a surgeon in his army.

Sir Astley had, for years, been subject to occasional attacks of giddiness, which he was naturally anxious to conceal, but which sometimes proceeded so far as to compel him to de-

* There is no truth whatever in the story which appeared in the newspapers some days ago, in which it is stated that Sir Astley lost his presence of mind on this occasion, and was recalled to himself by an admonition from Lord Liverpool; his Lordship was not even present.—Ed. Gaz.

sist from whatever he was employed about; and, on one occasion, he even fell in the street. During the latter part of his life he also suffered a good deal from occasional fits of the gout, which, however, did not present any thing remarkable. For some time his countenance exhibited a purplish tinge, as from some embarrassment in the circulation; and, latterly, he had been heard to protest, that he must decline visiting patients who were up two pair of stairs —a threat which, though meant as a joke, had evidently originated in the exertion distressing him. Indeed, we understand that he had repeatedly expressed his belief that there was "something wrong," and that he had not long to live.

The last and fatal attack, however, was of recent date, and in it he had the assistance of Dr. Chambers and Dr. Bright. The body was examined after death, when the diagnosis previously given was confirmed, by the heart being found to be enlarged, with some atheromatous deposits in the aorta, and effusion into the pericardium and cavities of the pleura.

The Chapel of Guy's Hospital has been appropriately chosen as the place of his interment, which is to take place to-morrow (Saturday) at three o'clock.

Sir Astley Cooper was a handsome man, and of striking appearance, well deserving the "c'est un bel homme!" which was often bestowed upon him as he walked round the Hôtel-Dieu with M. Dupuytren. His manner was open, free, and encouraging to his patients; altogether void of affectation, as well as of all excessive or artificial polish.

Throughout his whole career nothing could exceed the uniform fairness of his conduct towards all his brethren, or his kindness towards the younger members of the profession, especially those whom he saw to be possessed of merit. There are few among us who do not feel personally grateful to him for his conduct on some occasion.

Sir Astley, as we have seen, long enjoyed a large share of public patronage; but we believe the actual amount of his fortune, when stated at half a million, is considerably over-rated. His personal expenses were not great; but he was very liberal to his relations, on whom, we have heard, on what we believe to be good authority, that he bestowed between two and three thousand pounds annually. He is also said to have spent £20,000 in bringing his brother into Parliament.* Nor was his liberality confined to his own family:—when Dr. Baillie and some others made up a purse for Dr. Pemberton, in the difficulties brought upon him by his ill health, Sir Astley contributed the munificent sum of £500.

Besides his baronetage, Sir Astley had numerous honours conferred upon him: he was Serjeant-Surgeon to three successive monarchs: George IV., William IV., and her present Majesty. He received the Grand Cross of the Guelphic Order from his own sovereign, and the Cross of the Legion of Honour from Louis-Philippe. He was an honorary member of the French Institute, Doctor of Civil Law in the University of Oxford, and twice President of the College of Surgeons.

The leisure of his advanced age was not spent in idleness, but was devoted to scientific pursuits,—dissecting, making preparations, and other most industrious investigations of disease. We subjoin a list of his principal contributions to science—more honourable than all the dignities which have been bestowed upon him, and which will constitute the most enduring monument of his fame.

*List of Sir A. Cooper's Works and Papers.*— We believe that Sir Astley Cooper's first contributions to science are to be found in the *Medical Researches*, published in 1798. We therein find two papers,—

1. "The dissection of a case of hernia through the diaphragm, which proved fatal;" and

2. "Account of three cases of obstruction of the thoracic duct."

We next find him appearing in the *Philosophical Transactions* for 1800, to which he contributed

3. "Observations on the effects which take place from the destruction of the membrana tympani of the ear:" and in the same work for 1801, connected with the former paper, we have

4. "Account of an operation for the removal of a particular kind of deafness." For these he obtained the Copley medal.

5. In 1804 appeared "The anatomy and surgical treatment of inguinal and congenital hernia:" and in 1807

6. "The anatomy and surgical treatment of crural and umbilical hernia," which, thus completed, may be regarded as his great work, and that which first stamped his reputation with the strong impress which it ever after retained. As a treatise on the subject, it is still, and, perhaps, may always remain, unrivalled.

His next contributions appeared in the *Transactions of the Medico-Chirurgical Society*, the 1st volume of which contains

7. "Two cases of ligature of the carotid artery," being the first recorded cases of that operation; and the second volume has

8. "The dissection of a limb on which the operation for popliteal aneurism had been performed;" and also

9. "Some observations on spina bifida."

The 6th volume gives us

10. "The History of a case of premature puberty," and

* It is amusing to see Sir Astley's success attributed, in a memoir recently published, to his brother being in Parliament—just the converse being the fact, viz. that his previous success enabled him to make his brother an M. P.

11. "An Account of the anastomosis of the arteries of the groin," and the 8th

12. "Three cases of calculi removed from the bladder without the use of cutting instruments." In the 11th volume we have

13. "An Account of a case in which numerous calculi were extracted from the urinary bladder of the male without employing cutting instruments;" in the 12th volume is

14. "The History of an operation in which a fatty tumour, weighing above 37 lbs., was removed from the parietes of the abdomen;" and in the second part of the same volume is a

15. "Further account of the extraction of calculi from the bladder."

16. The next work we have to notice is the one containing surgical essays, by Sir Astley and Mr. Travers, in which the papers by the former are on Dislocations, on Exostosis, on Unnatural Apertures in the Urethra, on Encysted Tumours; and though last, not least, the case in which Sir Astley tied the Abdominal Aorta.

17. In 1822, the "Essays on Fractures and Dislocations" appeared as a distinct work, with numerous engravings; and, in 1823, an appendix was added, in reference to fractures of the neck of the femur. This is excellent as a contribution to science, while, as a text-book for students, it is invaluable.

18. "The Illustrations of the Diseases of the Breast" appeared in a quarto volume in 1829; and, in 1830, those

19. "On the Structure and Diseases of the Testis," in the same form, and also illustrated by expensive engravings.

20. Again, in 1832, we were presented with "The Anatomy of the Thymus Gland;" and in 1839 we have Sir Astley again,

21. "On the Anatomy of the Breast," constituting the last of his great works."

In the interval between the two volumes last mentioned, several papers were published in the *Guy's Hospital Reports*, besides some observations appended to the papers of others. Among his papers, given to the public through this channel, are two very interesting dissections, viz. "A Case of Femoral Aneurism, for which the external Iliac Artery was tied; with an Account of the Preparation of the Limb, dissected at the expiration of thirteen years;" and an "Account of the first successful Operation performed on the common Carotid Artery in 1808; with the post-mortem examination in 1821." Again, we have "Some Experiments and Observations on tying the Carotid and Vertebral Arteries, and the Pneumogastric, Phrenic, and Sympathetic Nerves;" again, we find a paper "On Spermatocele, or Varicocele of the Spermatic Cord;" once more we have a paper "On Dislocation of the Os Humeri upon the Dorsum Scapulæ, and upon Fractures near the Shoulder-Joint;" and lastly we have the "Dissection of a supposed Hermaphrodite." This is contained in the "Reports" for October, 1840; and is, we believe, the last of Sir Astley Cooper's numerous contributions to surgery.

We fear that Sir Astley's death has interrupted the completion of other works in which he was engaged. The second part of the "Illustrations of the Diseases of the Breast," which was to treat of malignant affections, was promised some time ago, but has not yet appeared. We hope that the materials which he collected for it will still be given to the public.—*Lon. Med. Gaz.*

---

*Lecture on Senile Gangrene.* By Sir B. C. Brodie.—Persons advanced in life are liable to mortification of the toes and feet; generally beginning in the former and extending to the latter. By persons advanced in life I mean those who bear upon them the marks of old age, which may, however, occur at various periods of human existence. One of the worst cases of mortification of the toes which I ever witnessed, connected with what might truly be considered old age, occurred in a man of six-and-thirty, worn out by the operation of bad habits upon an original bad constitution.

The question here arises, *in limine*, why is it that old persons are liable to this disease? Morbid anatomy enables us to answer this question. I have examined the bodies of a great many old persons who have died with mortification of the toes, and I have always found some morbid condition of the arteries of the affected limb. In the great majority of cases there is extensive ossification of the arteries of the thigh and leg. In many cases the arteries are not only ossified, but some of them are contracted and obliterated. Thus I have known the femoral artery to be obliterated from the origin of the *profunda* down to the ham. In other cases one or more of the arteries of the leg are obliterated, while the femoral artery is still pervious. In one case, of which I have preserved notes, the arteries were not ossified in any part of their course, but the femoral artery was converted into a gristly cord, and quite impervious from the origin of the profunda to the point at which it perforates the tendon of the great head of the triceps adductor muscle. In none of these cases in which the arteries were contracted and impervious, were there any such appearances as would have indicated that the contraction had been the result of previous inflammation; and it appeared to me that the change which had taken place in their condition was best to be explained by supposing it to be the result of a process corresponding to that which produces stricture of the urethra or œsophagus.

It has been said that mortification of the toes in old persons is often the result of disease in the heart itself. This does not, however, exactly correspond with the results of my own experience. It is true that I have known persons who had disease in the heart, to die of mortification of the toes; but then there was always enough in the condition of the arteries of the limb to account for the mortification, independently of the other disease. Thus, in one case in which there was mortification of the right foot, the muscular structure of the heart was soft, thin, flaccid, and easily torn; one coronary artery was impervious; and the right iliac artery, for the extent of three inches, was impervious also, in consequence of it being completely filled by a mass of firmly coagulated blood. In another case, in which there had been mortification of the right foot, the muscular structure of the heart was pale and flaccid; one coronary artery was contracted and impervious; the cavities were dilated; a mass of dense coagulum, resembling that found in the sac of an aneurism, occupied the appendix of the left auricle, and there was a similar coagulum obstructing the popliteal artery and vein of the right side, and extending some way down the branches of those vessels in the leg.

You are not, however, to suppose that mortification of the toes is a necessary consequence of ossification or obliteration of the arteries, and that it occurs in all such cases. I have no doubt that many persons have the arteries thus altered in structure for many years, although mortification never supervenes. I have already explained to you that in some cases the arteries are ossified, and at the same time either contracted or obliterated; that in others they are obliterated without being ossified, or ossified without being obliterated, even retaining their natural diameter. It is evident that the quantity of blood admitted into the limb must be different in these different cases, and that the liability to mortification must vary accordingly. But further than this: even where arteries are rendered narrower, or actually obliterated, it seems that in general something more must happen to bring on mortification; and you will almost invariably find that the immediate cause is an attack of inflammation. Perhaps the following is not an unreasonable explanation of the phenomena which occur. The arteries are ossified, or they are partially obliterated; but still a sufficient supply of blood for ordinary purposes, goes to the limb. By and by, from some cause or another, the foot becomes inflamed. I observed to you, in a former lecture, that during inflammation an increased supply of arterial blood seems to be required, and that the arterial trunks leading to the inflamed part become dilated, so as to allow this increased quantity of blood to enter; but if the arteries are ossified, they lose the power of dilatation; they cannot expand; the greater supply of blood required in consequence of the inflammation is withheld, and so the part perishes.

You might suppose, *à priori*, that persons in the lower condition of life, who live hard by their daily labour, would be more liable to mortification of the toes than other persons; but such is not the case; at least it has fallen to my lot to see comparatively few cases of this disease in the hospital; whereas, in private practice, I have met with a great number; so that for one case under my care in the former I have had three or four in the latter. It is one of the penalties paid by those who enjoy the advantages of ease and affluence, and who live luxuriously. It is persons who eat too much, and drink too much fermented liquor, and do not take sufficient exercise, that are especially liable to this disease, and not the labouring poor.

Ossification of the arteries is a change that can take place only gradually; and the obliteration of those vessels which I mentioned as occurring in some cases, probably takes place gradually also. You will easily believe that, under those circumstances, certain premonitory symptoms may arise in the lower limb before the disease is gone so far as to produce mortification. If you cross-examine a patient who has mortification of the toes, he will generally tell you, that for three or four years preceding he has had occasional pains in the lower limbs; a sense of numbness in them; that his feet were liable to be cold; that when they again become warm, after having been cold, they have been very painful; and that he has had a sense of weakness of the muscles. Such patients walk a short distance very well; but when they walk further the muscles seem to be unequal to the task, so that they cannot get on. The muscles are not absolutely paralyzed, but in a state approaching to it. All this is easily explained. The lower limbs require sometimes a larger and sometimes a smaller supply of blood. When more blood is wanted, the arteries cannot open to let it in, and hence arise both pain and numbness. In walking, the muscles ought to receive an increased supply of blood, but, the arteries being ossified or obliterated, they are incapable of transmitting it; and this explains the sense of weakness. This last circumstance may be illustrated by what you observe in a particular disease of the heart. Dr. Jenner first, and Dr. Parry, of Bath, afterwards, published observations which were supposed to prove that the disease which is usually called *angina pectoris* depends on ossification of the coronary arteries. I will not say that such symptoms as those of *angina pectoris* can arise from no other cause, but I know that they do arise from it in certain instances. In two cases in which I examined the bodies of persons who died from the disease in question, I found ossification of the coronary arteries to a great extent, so that they were converted into complete bony tubes, while there was no disease of any consequence besides. When the coronary arteries are in this condition, they may be capable of admitting a moderate supply of blood to the muscular structure of the heart, and so long as the patient makes no unusual exertion, the circulation goes on well enough. When, however, the heart is excited to increased action, whether it be during a fit of passion, or in running or walking up stairs, or lifting weights, then, the ossified arteries being incapable of expanding to let in the additional quantity of blood which, under these circumstances, is required, its action stops, and there is syncope; and I say, that something like this may be observed in persons who have ossified or obstructed arteries of the legs.

These premonitory symptoms, as I have said, may exist for three or four years, until at last some accidental attack of inflammation occurs which induces the mortification. A very frequent occurrence is this: the patient cuts a corn, the knife goes below it, makes the toe bleed, and a little inflammation follows: or it may be, that the foot gets chilled by exposure to cold, and the patient goes to the fire to warm it, and that this is followed by a degree of inflammation which, if the arteries were healthy, would be a chilblain and nothing more, but which, in their present condition, lays the foundation of mortification. A slight degree of inflammation of the toes almost invariably precedes the mortification; vesications then take place, the vesicles burst, and at the bottom of them you find the cutis to be dead. This may take place in one toe, or in many toes at the same time. Most frequently, the disease having commenced in one toe, extends to the others, and then to the feet. Frequently, in the beginning of the complaint, there is a most intense pain, but sometimes the pain is very trifling. The mortification having once begun, a little inflammation is kept up on its margin, which slowly creeps up the foot, and the mortification follows it; the constitution being probably little or not at all disturbed, the pulse remaining at its natural standard, and the patient in all other respects thinking himself well. The disease, in fact, generally has, in the first instance, a chronic form; but sometimes it is otherwise, so that it exhibits all the characters of an acute disease. The man to whom I before alluded as old in constitution, though not in years, being only thirty-six, had been a soldier, and had served in Canada and in the East Indies—that is, in cold climates and in hot. He had, by his own acknowledgment, been a drunken fellow, and dissipated in other ways. Having been dismissed from the army as superannuated, he gained his livelihood by working as a labourer in the Edgware Road. Many times, on going to work, he suffered from cold and numbness of the feet, followed by violent pain. One morning in September (not a very cold time of the year) these sensations took place to a very great extent; severe pain and shivering followed, and his friends took him home in a coach. Two days afterwards he was brought to the hospital; and then all the toes of one foot were mortified, and one or two of the other. Under the treatment which was employed, and which I need not explain at this moment, he recovered. The dead toes came away, the sores healed, and he left the hospital as cured. Two years afterwards he was re-admitted with an abscess on one inststep, and a sinus running under the skin. This occurred the year after I had been elected assistant-surgeon to the hospital; and not knowing any better at that time, I introduced a director under the skin and along the sinus, and, according to what I had been taught to do in a case of this kind, I slit open the sinus with a lancet, making an incision two inches in length. With my present knowledge I should have acted otherwise. Some inflammation followed the wound which extended to the foot. The next day mortification had extended up the whole foot to the leg, the pulse was frequent and weak, the skin hot, and the patient lay in a state of stupor. Two days afterwards he died. You will observe that in each of these attacks the disease had the acute form, and that in the second attack it terminated life in about four days. I examined the body after death, and found extensive ossification of the arteries of both limbs.

The more common history of the disease, however, is this: in its origin it has the chronic form, but if it goes on, it sooner or later assumes the acute form. The mortification may gradually spread up the toes and feet without any urgent symptoms, and this may be going on for weeks, and even for months; then, all at once, a fresh attack of inflammation takes place, the mortification extends rapidly, the constitution suffers, the pulse becomes feeble and rapid, the patient falls into a state of stupor, and dies in the course of a few days.

There is no form of mortification which is more dangerous than that of which I am now speaking. A large proportion, indeed, of the patients who are so affected, under any mode of treatment, die. You will not be surprised, then, that a great many different modes of treatment have been proposed. Where there is a disease that always gets well under a certain system, medical men have little inducement to make experiments; and the wisest make none at all. But in an intractable disease like this it is natural that practitioners should be always looking out for new remedies. I do not pretend to speak of all the variety of remedies that have been used or recommended; but I shall allude to the principal ones.

In the first place, those who have observed that the disease is preceded by inflammation have said, "bleed the patient; treat it like an inflammatory disease." I have no doubt that some have been led to recommend this from a mistake respecting the pathology of this disease, which I noticed in the last lecture; that is, from having supposed that this peculiar kind of mortification of the toe depends on inflammation of the arteries. I have, however, explained to you that the two cases are quite different. Bleeding has, however, been proposed, and in one instance I saw it tried. The mortification was to a very small extent; there was but very little inflammation round it, and the patient seemed to have a very fair chance of recovery. But immediately after the bleeding the mortification extended rapidly up the foot, and he died. Indeed, it appears to me that we have no right to expect that we shall cure this disease by taking away blood. There is inflammation, it is true; but if the inflammation terminates in mortification, it is because the part, on the principle which I just now explained, cannot get that additional supply of blood which an inflamed part requires. Now, if you abstract blood, and thereby lessen the quantity in the system and weaken the action of the heart, the supply of blood to the limb must be diminished, and the cause of the disease aggravated.

An opposite plan of treatment to this has been recommended by others. They have said, "this is a disease of weakness; give bark, quinine, serpentaria, and other tonics." Now there are certain kinds of debility which will be relieved by these remedies, but here there is only a local weakness, depending on disease of the blood-vessels. Will such remedies as these mend the condition of the arteries? Certainly they will not; but they will interfere with the digestion; they will prevent so much food from being converted into nourishment as would be converted into it otherwise; they will prevent the exhibition of stimulants which really are useful, as I shall explain presently. I own that I have very little, I may almost say no faith, derived either from theory or from practice, in the good supposed to be produced by the exhibition of what are called tonics. If you give anything of the kind, let it be ammonia, combined with the compound infusion of orange peel. Ammonia for a little time may be useful; but I think that there are objections to its long-continued use in this and in every other case. It appears to me that patients who take it for a long time are at last rendered weaker by it instead of stronger. It is an alkali, and produces the same effect on the blood that is produced by other alkalies. If it be taken, however, for a short time, it may be useful.

In the management of these cases, there can be no doubt that one principal object to be kept in view is the maintenance of a sufficient supply of blood in the system. As the abstraction of blood is mischievous, so the opposite treatment is likely to be beneficial. Let the patient, then, be put on a system of nutritious diet, not overloading his stomach so as to produce a red or yellow sediment in the urine, but taking as much food as can be easily assimilated, and no more. Let him live chiefly, but not entirely, on animal food, which makes blood—if I may use the expression—of a better or stronger quality than that derived from vegetables alone. In addition to this, the patient will require the use of some such stimulants as ale, wine, or brandy. You will generally find that persons who have mortification of the toes are such as have been accustomed to take a good deal of fermented or spirituous liquor, and being accustomed to it, that they cannot do without it. Nor is this all. Those whose mode of life has been different will require the exhibition of stimulants under these new circumstances. The question, however, will arise in each individual case, what is the proper quantity to be exhibited? Some persons may want a bottle of wine daily; but very few, on this or on other occasions, are benefitted by so large an allowance as this. In the majority of cases, from half a pint to a pint daily will be sufficient. You should ascertain what have been your patient's previous habits, and then give him wine or ale cautiously, observing the effect produced. There is one good rule of conduct in this respect, both in health and in disease: wine that does not occasion heat of skin, that does not raise the pulse, nor make the mouth clammy, nor render the patient nervous or irritable, any quantity that does not produce these effects, may be given with advantage; but otherwise it does mischief.

In all cases of mortification of the toes, I have observed it to be of great consequence to attend to the state of the digestive organs. If the bowels are not in a proper state, the food cannot be properly assimilated; and the patient being confined, as he must be, to his bed, the bowels will not act without assistance. I do not advise you to give purgatives every day, but rather an active dose may be required once in three or four days; such as two or three grains of calomel at bed-time, with an aperient draught on the following morning, or blue pill with compound extract of colocynth; and all my experience leads me to believe that this is a very essential part of the treatment.

Mr. Pott was either the first who recommended, or the first who brought into general use, the exhibition of opium in cases of senile gangrene. What is the *modus operandi* of opium here I will not pretend to say; but I can have no doubt, from all the experience that I have had, that there is really no internal remedy so useful as this. I can scarcely remember meeting with a single case of recovery in an old man, from mortification of the toes, in which opium had not been exhibited. But it is with opium as with wine; a good deal of discretion is necessary as to the exhibition of it. You must not begin with very large doses of opium; they are too powerful for the constitution, and opium is mischievous if it keeps the patient dozing all the day. You may at first exhibit half a grain three times daily, and keep him slightly under its influence, but nothing more. If he continues to take it (and sometimes this may be necessary for months together,) the dose will require to be increased; but you will never be able to persevere in the use of opium, except you employ in combination with it those remedies which I last mentioned. Not only purgatives, but mercurial purgatives, are required by all persons who take opium in this manner, otherwise it stops the secretion of bile, and does mischief. The result of the case will very much depend on this—whether opium does or does not agree with the patient. If opium induces a feverish state of system, if it disturbs the sensorium, if it interferes in any way with the digestion of the food, and especially if it makes the tongue brown and dry, it can do no good; while the mere healthy action of it will be almost certainly beneficial.

With respect to the local treatment, the first thing is to keep the patient in bed. Not feeling very ill, he probably will wish merely to lie on the sofa; but this never answers; therefore send him to bed at once. If he strives against it for the first few days, he will be driven to bed at last, and will be worse than if he had gone there in the first instance. I think a great deal of the success of the treatment will depend on his being placed in the uniform warmth of bed at the very commencement of the attack. Rest in bed in the recumbent posture is essential. Then, what local treatment is required besides? It is common to apply poultices made of grounds of stale beer, or of red wine and oatmeal, and some recommend a solution of chloride of soda. I was accustomed formerly to rub the legs and thighs with a stimulating liniment, but I soon left off this practice, finding that it did no good; and I believe now that, if it does anything, it does harm. Why do the toes mortify? Because when inflamed they do not get a sufficient supply of blood. Rub the thigh and leg with a stimulating liniment, and it is the same thing, only in less degree, as blistering them: and what would be the consequence of applying blisters? It would draw the blood to another part. You want it in the foot, and you draw it elsewhere. It is something like taking blood from the arm, not indeed so mischievous: less in degree, but the same in kind. Then, I must say, that I have never seen any good from it in practice. Neither have I any reason, from what I have seen, to believe that those other applications which I have mentioned, used as poultices and lotions, are of any use.

Some few years ago, I was in consultation with the late Mr. Vance, of Sackville street. He had been surgeon for many years to Greenwich Hospital. Being always anxious to obtain what information I can from others, I observed to him, "You must have seen among the old men at Greenwich a great number of cases of mortification of the toes. What have you found, on the whole, to be the best local treatment?" He answered, that he had found nothing to answer so well as wrapping up the parts in carded wool. I did not understand from him whether he wrapped up merely the foot or leg, or the whole limb; but he added that he usually left it on for many days. It struck me that this was a very reasonable kind of practice. Wool is a very bad conductor of heat, and wrapped round a limb it must keep it of very uniform temperature, and at any rate save, in a great degree, expense and trouble of generating animal heat. Soon afterwards I had an opportunity of adopting Mr. Vance's mode of treatment. I had been poulticing a foot as usual, and the disease was going on spreading from one toe to another, and up the foot. Carded wool is so prepared that it may be drawn out in long flakes several feet in length, and in these I wrapped up the foot; and then, thinking that I had better proceed further, I wrapped up the leg and the thigh also, as high as the middle of the thigh. I applied it rather loosely, one flake over another, until the limb appeared to be three or four times more bulky than it was in its natural state. The result was excellent. The mortification never spread from the time that the wool was applied, and the patient recovered. I have employed the same local treatment since in other cases, and although, of course, it would be absurd to represent it as always successful, yet I feel bound to say that I am satisfied that it produces much better results than any which I have ever employed.

In employing the wool, recollect that you should apply it loosely and uniformly, and plenty of it. You may afterwards sew it all up in a silk handkerchief, and leave it unopened for several days, sometimes a week. You may lay a simple dressing of calamine cerate on the mortified parts, replacing it whenever you change the wool. If the mortification stops, and the slough is coming away, you may, on account of the discharge which takes place, change the wool every other day. The carded wool possesses, as a little consideration will prove to you, many advantages over the poultices. In the first place, if you use poultices the limb is exposed alternately to cold air and hot poultices three times every twenty-four hours, that is, to repeated changes of temperature. In the intervals it is at any rate left to generate heat as usual. But if you wrap it up in carded wool, both these things are avoided. In another respect, also, this mode of treatment is a great comfort to the surgeon, the patient, and the whole family. Two or three times daily, whenever the poultices are changed, the family inquire, "Is he better? is he worse? is the mortification stopped?" You are called upon to answer these unanswerable questions, and the patients' mind is kept in a constant state of excitement. But if you put on the carded wool, and leave it there, his mind in the interval is tolerably tranquil: he lives upon the hope that, when the wool is next taken off, the parts will be found better; and such a state of mind is much more favorable to his recovery than the nervous anxiety which he experiences when the limb is examined more frequently. I believe that there are very few cases to which you will not find this method of treatment applicable. If there be any, it is those in which there is great inflammation and heat of skin, and in these it may be prudent to defer the application of the wool until these symptoms are abated.

Whenever the mortification is arrested, you will be made aware of it by a line of separation on the margin. The process of separation proceeds, in favourable cases, until the bones of the toes come away. You may have to cut through some dead ligaments and tendons, in order to promote the separation of the offensive and putrid parts, but you must cut through nothing else. If you apply your knife to living parts, you will certainly bring on a fresh attack of mortification. Leave the separation altogether to nature, and the natural process will do all that is required.

But there is another question. A man has mortification of the toes, and, independently of experience, you might naturally say,—here is a most dangerous disease; why not at once amputate the limb? It is probably unnecessary for me to tell you that it would be contrary to all the old rules of surgery (for which I have great respect) to amputate a limb under such circumstances. I have never seen it done: I have never done it myself, but I have heard of cases in which the surgeon was, shall I say fool enough or ignorant enough? to venture on this summary proceeding of cutting off the leg, because the toes were beginning to mortify. In every instance the stump mortified directly, and the patient died. The chance of recovery from mortification of the toes is not very considerable—that is to say, there is a great chance of the patient dying; but still, under proper treatment, there is also a fair chance of recovery, and you ought not to risk this chance by inflicting on the diseased limb so severe a local injury as belongs to amputation.

I have told you that disease of the arteries lays the foundation of mortification; but the disease may exist many years without mortification supervening, until some accidental circumstance brings on inflammation. I have known persons with disease of the arteries, and several toes mortified in consequence of it, in whom the mortification has stopped, the sloughs have separated, the sores have healed, and who have lived for years afterwards. I know a gentleman who is now alive, and in good bodily health, at least he was so not long since, whom I attended for mortification of the toes nearly five years ago. This patient was treated on the carded wool plan, and I cannot but suspect that it did something more than relieve the disease at the time. At all events, it may be admitted as a question, whether the keeping the limb wrapped up in the carded wool, which is like keeping it in a vapour bath, may not ultimately produce some beneficial change in the condition of the diseased arteries; not indeed removing the phosphate of lime, which is deposited in their structure, but leading to their becoming gradually and slowly expanded, so as to allow of a more liberal supply of blood to the limb. Whether this suspicion be or be not well founded, I suppose that no one will doubt that it will be prudent in all cases to advise the patient after his recovery, always to wear a thick fleecy hosiery stocking, or to use some other kind of warm clothing, so as to preserve the limb from the influence of the external cold.

I must add a very few words respecting the treatment during the process of separation of the dead parts. Bark, quinine, and other tonics, may be useful now, though they were not so before. Wine, and a generous diet, are still required; and some stimulating dressings, such as the unguentum elemi compositum, may be useful applications to the sores.—*Ibid.*

---

*Rare Malformation of the Heart.* By Albert Napper.—A. W., ætat. five years and ten months, a lively, intelligent boy, but rather short of his age. Till he was six months old his mother observed nothing unusual in him, except that his head was rather larger than natural. About that time he received a fall, after which he was affected with cyanosis, and was from that time always subject to dyspnœa, and palpitations of the heart, greatly increased by exertion and mental emotions, as was also the purple hue. He had had measles, whooping-cough, and small-pox; the latter very severely.

A short time since, I was requested to see him, when he complained of pain in the head, accompanied with drowsiness, pyrexia, and other symptoms of acute hydrocephalus, which quickly carried him off. On examination of the brain, extensive ramollissement of the right hemisphere, and serous effusion, was found to exist.

Before describing the formation of the heart, which is one of those singular instances of monstrosity but seldom displayed by nature, I must mention a peculiarity, doubtless in connection with it, exhibited by the lungs, which were extremely small and flaccid, and of a bright crimson throughout, but evidently not the effect of inflammatory action, for no other indication of inflammation was present, nor was there any thing during life which at all led to a suspicion of its existence; on the contrary, in expressing a wish to be carried down stairs, he spoke so loud and distinctly, that his mother, who was below, heard every word. His wish was complied with, and within three minutes he was a corpse.

On opening the pericardium, the heart was seen of normal dimensions, with both ventricles contracted, and *both* auricles extremely congested with dark-coloured blood. The parietes were of usual thickness, and the valves natural. In the situation of the foramen ovale was an opening, about half an inch in length and a line in breadth, with a band of fibres extending across the centre, attached to either lip. Judging from the valvular form of the opening, it is probable that no blood passed through it, except perhaps during the impetus of the circulation from excitement. But another and more remarkable deviation was exhibited in the aorta. The orifice, instead of its usual commencement from the left ventricle, was placed directly over the septum ventriculorum, communicating *equally* with *both* ventricles. The ventricular orifice of the pulmonary artery was extremely contracted, scarcely admitting the large end of a common silver blow-pipe. At a short distance from the origin, the artery became much larger, but had more the appearance of a vein. The sigmoid valves were exceedingly small, but otherwise perfect. From the situation and contracted orifice of this vessel, and from the ready passage of the blood into the aorta, it is evident that but a very small portion of blood could have passed to the lungs; which may account for their being so much below the usual size. Considering the very small quantity of arterialized blood that could have entered into the circulation, it is surprising that this child should have attained the age of nearly six years, have passed through some of the worst diseases of childhood, and that the functions of the system, excepting those above mentioned, should have been naturally performed.

P. S.—There is a case somewhat similar to the preceding, related in the Medico Chirurgical Review for July, 1831, p. 211.—*Ibid.*

---

*Operation for the Cure of Wry-Neck.* By Henry Symes, M. R. C. S. L.—Jane Irish, a delicate girl, aged sixteen, was the subject of congenital contraction of the right sterno-cleido mastoid muscle, and, on examination, the following appearances presented themselves:—The head was closely approximated to the shoulder, the right mastoid process lying within two inches of the clavicle. The face was drawn to the opposite side, looking obliquely upwards. The right clavicle was extremely convex, and seemed as if pulled upwards; the right shoulder was raised at least three inches higher than the left; the trapezius muscle of that side being exceedingly prominent, forming, when the head was bent to the opposite side, a large ball. The deformity, (as will appear,) was not at all connected with any spinal affection, for, on tracing the spinous processes of the vertebræ, they were found to preserve their normal direction. There was a lateral curve in the cervical region, but this depended *solely* upon the contracted state of the muscle. Being satisfied, from this examination, that the spine had no share in the deformity, I determined on a division of the muscle, which I performed after the following manner, and which has been, on several occasions, so successfully performed by Professor Dieffenbach, of Berlin.

The patient being placed in a chair, I caused the head to be drawn to the opposite side by one assistant, whilst a second depressed the shoulder of the affected side. The muscle being thus rendered more prominent, I passed a narrow curved bistoury about an inch above the clavicle, behind the muscle, dividing both its origins as I withdrew the knife, without causing any further wound of the skin, when scarcely a drop of blood followed. At the moment of the division there was an audible sound; and the head not only became immediately erect, but the prominence also greatly diminished, and the shoulder itself fell at least an inch. A dossil of lint was firmly applied by adhesive plaster, so as to prevent extravasation, or if any, by its pressure to cause its speedy absorption. The patient, who seemed to suffer but slightly from the operation, was then sent to bed, where she remained until the following Friday, during which time the head was kept in its new position by the use of bandages. On the removal of the lint, &c, (which had not been previously disturbed,) I found the wound quite healed, and there was not even the slightest discoloration of the skin. I now applied a stiff pasteboard collar, in such a manner as to oblige the patient to incline the head to the opposite side, which served to elongate the callus. By the use of straps, and by confinement to an inclined plane during a greater portion of the day, for a fortnight, the prominence has nearly disappeared. The shoulder is now on a level with its fellow, and the head is perfectly upright; so that, to an ordinary observer, the patient presents no vestige of her previous deformity.—*Lon. Med. Gaz.*

---

*Case of Scirrhus of the Optic Thalamus and Corpus Striatum.* By John Waters, M. D.—In the month of September, 1837, I was called to see a gentleman, 33 years of age, of robust constitution, who was seized with a fit, apparently of an apoplectic nature. There was complete annihilation of the intellectual and locomotive powers; the expression of the face was vacant, and the complexion was of an exceedingly yellow straw-colour. There was slight strabismus divergens of the left eye, and the pupils of both were much dilated; there was no spasmodic constriction of the maxilla, extremities, nor frothing of the mouth. Respiration was regular, though protracted, and occasionally accompanied with a few forcible and deep expirations; the heart's action was labouring, slow, (60,) and the impulse of the left ventricle was sensibly felt by the hand at the precordial region, accompanied with marked *bruit de soufflet*, which could be even easily distinguished in the carotids; percussion dull in a circumscribed portion of the left side of the heart's region. There was an involuntary discharge of fæces.

He remained in this comatose state for about twenty minutes, when he gradually resumed all his faculties, though still feeling some sensation of prostration. I then learned that this was the tenth attack which he suffered from during a period of nearly two years and a half; that he generally complained of violent headach; and had a sensation as of some foreign body in his brain, which induced him frequently to shake his head; he never presented any symptoms of spasmodic contraction, or paralysis in any organ; memory not impaired; appetite good; bowels regular. Until the last twelve months he led rather an intemperate life, but cannot even attempt to resume the slightest irregularity; was much addicted to venereal excitement. He, as well as the other members of his family, assured me that his father died (in their opinion) of an affection similar to the one which I was called to see; the mother, whom I myself remember, died suddenly, although apparently in the enjoyment of health. Ordered him to take at once, calomel, five grains; tartar emetic, quarter of a grain; extract of conium, two grains; carraway oil, two drops; and in three hours to have two large spoonsful of the following mixture: compound infusion of senna, two ounces; camphor mixture, three ounces; sulphate of magnesia, four drachms; dilute sulphuric acid, two drachms; orange-flower water, half an ounce; syrup of rhamnus, two drachms.

On calling next day, Sept. 21st, I found that his bowels had been well acted on; he slept well; pulse 70, regular, full, and strong; tongue clean; no thirst. The strabismus of the left eye continues since the fit, and he complains of slight dimness of vision, and a tottering condition of the lower extremities, such as may be felt after a day's severe exercise; the mouth for the first time now presented a slight deviation; has no recollection of the preceding day, and feels an indescribable uneasiness in his head. Ordered to be cupped from the lumbar region to sixteen ounces; mustard sinapisms; blue pill, twelve grains; digitalis, six grains; compound calomel pill, twenty grains; extract of conium, twelve grains; divide into twelve pills, one every second hour; milk diet.

22. The strabismus improved; head not so troublesome; bowels relieved; pulse more compressible; the debility felt in the lower extremities much less; feels much better. Treatment continued. He went on each day improving, and on the 29th was sent to the country, when there was no strabismus, scarcely any deviation of the mouth, and his feelings of strength quite recovered. I saw him occasionally afterwards; and early in the following December, I met him accidentally, but not at his own house. He then complained of great confusion of ideas, loss of memory, which was so great that he could not recall what had passed a few minutes before; vision was considerably impaired, and under the influence of the strongest light the pupil remained much dilated; could give little or no account of his sensations; the heart's action was not so forcible as usual, regular. Desired him to go home, whither he was accompanied by a servant, who left him at his own door. I afterwards learned that on the door being closed he fell, but immediately recovered himself and went up stair, but not to his own room, remarking that he was completely blind. In the morning he was found dead in bed.

*Autopsy twenty-five hours after death.*

Cadaveric rigidity considerable; the external appearance presented nothing worthy of note, except some spots of ecchymosis on the face and chest. On dividing the scalp there was a great effusion of blood; the vessels of the pia mater were very turgid, but there was no effusion of blood on the surface or in the convolutions; the superficies of the brain was rather moist. On slicing the hemispheres the same state of moisture was observed, without the slightest trace of injection; the most minute search was made for chronic apoplectic cysts, but without success; both ventricles were filled with a fluid of a light yellow colour. The choroid plexus on the left side was in an œdematous condition; and I found a few small concretions of the size of a millet-seed, in the left. The optic thalamus of the left side presented a central spot of ramollissement, without the slightest trace of vascularity. The corpus striatum presented nothing abnormal. The corpus callorum and fornix in a normal state; the third, fourth and fifth ventricles also contained a similar fluid to that in the lateral ventricles. The right ventricle was also filled with yellowish coloured fluid; the choroid plexus was remarkably pale, and by no means œdematous. On examining the corpus striatum and optic thalamus of this side, their surfaces were completely covered with a semi-fluid pultaceous matter, of a dirty grayish colour. On removing this partly liquefied mass, there was a central nucleus which filled each organ, of a semi-transparent glossiness, exceedingly hard and dense, without a trace of vascularity, and had become more adherent to the base of the ventricle than in the normal state. Cerebellum presented nothing unusual. On dividing these indurated masses, they had an almost cartilaginous consistence, were of a bluish aspect, and were traversed by fibrous septa; the substance of the base of the brain presented nothing unusual, but there was a great effusion of the sero-sanguineous fluid, which evidently came from the spinal canal, for as quickly as it was removed, it was visibly replaced by a continual flow from that source. There was no trace of hyperæmia. On examining the spinal column, it was found distended with a bloody fluid, and the vessels were evidently in a congested state. The substance of the cord presented nothing remarkable; there was no coating of either a fibrinous or albuminous nature. The lungs were healthy, if we except a few ancient adherences on the right side. The pericardium contained about a dessert-spoonful of serum; and viewing the heart in situ, its volume appeared much increased, particularly the left ventricle, which was considerably developed; its cavity was much diminished, constituting that form of hypertrophy denominated "the concentric," by M. Bertin. The carneæ columnæ also entered into the state of hypertrophy. The aorta and pulmonary vessels healthy. Abdomen not examined.

*Remarks.*—The case here reported is one of exceedingly rare occurrence. M. Andral, in his *Clinique Médicale*, does not relate a single case of scirrhus of the organs affected in my patient. When we consider the extensive disorganization of two such important organs, we are naturally led to conclude that its effects on the system should have been well marked; but there was no one single symptom to elucidate a lesion of the importance just related; and though some late physiological experiments, from which certain functions are assigned to the different organs of the brain, are apparently conclusive, it shows how guarded we should be in forming conclusions that are not borne out by pathological research,—for in the case before us, there was no lesion of the muscles of animal life. M. Cruveilhier, speaking on this subject, says, "that lesions of the fibrous substance and central parts of the brain govern voluntary movements; that lesions of the optic thalamus, and its radiations, regulate the movements of the upper extremity, the corpus striatum those of the lower. The alteration of the corpus striatum and optic thalamus, considered together, and abstraction made of the special structure of each, produces the same effects as the alteration of all the fibrous substance of the hemispheres, of which they are in some manner the centre." The only constant symptoms which attracted attention were the incessant headach, and frequent attacks of an apparently apoplectic nature, for which he had been always treated before I saw him; and even then there was more reason to suspect the irritation resulted from an ancient apoplectic cyst, than a fresh attack of apoplexy. There was a peculiarity in the complexion of the patient, which might have called attention. The immediate cause of death was a sudden and immense effusion into the spinal canal. The effused fluid in the ventricles was of a longer date, and evidently the result of the irritation produced by the morbid growths; the brain became, as it were, accustomed to this gradually increased compression from the length of time of their development. There is another important feature to which I wish to call attention,—the hereditary tendency which I presume existed in this case, for the gentleman's father and mother died from an affection which was called apoplexy; and some short time after the death of the individual who forms the subject of the present history, his youngest sister, who was very subject to severe headach, fell in the street, and from her account it was not by accident, as she afterwards allowed she felt quite insensible, and was unconscious of such an occurrence.—*Provincial Medical and Surgical Journal.*

---

*On Effusions into the Cavity of the Thorax, and a new method of evacuating them.* By M. Reybard.—The new method proposed by M. Reybard for the evacuation of fluids effused into the cavity of the thorax is, "to leave the opening which may be made in the thorax free during each act of expiration, but to close it during each inspiration," and thus prevent the ingress of air into the thoracic cavity, to which he attributes the numerous failures of the operation for empyema, &c. For this purpose, he introduces into the wound a tube or canula, to the extremity of which is attached a portion of cat's intestine, softened by immersion in warm water, about three inches long, and open at both ends. During expiration, this instrument gives free passage to any pus or fluid from the cavity of the thorax; but during inspiration the sides of the gut collapse, and, acting as a valve, completely prevent the air from entering the thorax.

Having performed several experiments with his valved canula on animals, and acquired a conviction of its utility, the author employed it in several cases of empyema and hydrothorax, four of which he describes at considerable length. In one case, the patient was cured on the fifteenth day; in another, on the thirty-fifth day; in a third, it required four months of constant care before the wound in the thorax was completely healed.—*Ibid.*

---

*Employment of Hydrated Peroxide of Iron as an antidote for Scheele's green.* By Dr. Spaeth, of Esslingen.—An infant three years old, having swallowed a spoonful of Scheele's green, (arsenite of copper,) very soon became attacked with violent vomiting, diarrhœa, violent pain in the belly, and insatiable thirst. He was in the first instance made to drink cold water, and then fifteen grammes of the peroxide of iron were administered to him, suspended in hot water. He took four doses of it.

An hour after the employment of the antidote both the vomiting and the diarrhœa had ceased, as well as the pain and the thirst; the next day all the symptoms of poisoning had ceased.

*Gazette Medicale.*

